# DeepPVP: phenotype-based prioritization of causative variants using deep learning

**DOI:** 10.1186/s12859-019-2633-8

**Published:** 2019-02-06

**Authors:** Imane Boudellioua, Maxat Kulmanov, Paul N. Schofield, Georgios V. Gkoutos, Robert Hoehndorf

**Affiliations:** 1 Computational Bioscience Research Center (CBRC), King Abdullah University of Science and Technology, 4700 KAUST, Thuwal, 23955-6900 Kingdom of Saudi Arabia; 2 Computer, Electrical and Mathematical Sciences & Engineering Division (CEMSE), King Abdullah University of Science and Technology, 4700 KAUST, PO Box 2882, Thuwal, 23955-6900 Kingdom of Saudi Arabia; 3 Department of Physiology, Development & Neuroscience, University of Cambridge, Downing Street, Cambridge, CB2 3EG UK; 4 College of Medical and Dental Sciences, Institute of Cancer and Genomic Sciences, Centre for Computational Biology, University of Birmingham, Birmingham, B15 2TT UK; 5 Institute of Translational Medicine, University Hospitals Birmingham, NHS Foundation Trust, Birmingham, B15 2TT UK; 6 NIHR Experimental Cancer Medicine Centre, Birmingham, B15 2TT UK; 7 NIHR Surgical Reconstruction and Microbiology, Birmingham, B15 2TT UK; 8 NIHR Biomedical Research Centre, Birmingham, B15 2TT UK; 9 MRC Health Data Research UK, Birmingham, B15 2TT UK

**Keywords:** Variant prioritization, Phenotype, Machine learning, Ontology

## Abstract

**Background:**

Prioritization of variants in personal genomic data is a major challenge. Recently, computational methods that rely on comparing phenotype similarity have shown to be useful to identify causative variants. In these methods, pathogenicity prediction is combined with a semantic similarity measure to prioritize not only variants that are likely to be dysfunctional but those that are likely involved in the pathogenesis of a patient’s phenotype.

**Results:**

We have developed DeepPVP, a variant prioritization method that combined automated inference with deep neural networks to identify the likely causative variants in whole exome or whole genome sequence data. We demonstrate that DeepPVP performs significantly better than existing methods, including phenotype-based methods that use similar features. DeepPVP is freely available at https://github.com/bio-ontology-research-group/phenomenet-vp.

**Conclusions:**

DeepPVP further improves on existing variant prioritization methods both in terms of speed as well as accuracy.

**Electronic supplementary material:**

The online version of this article (10.1186/s12859-019-2633-8) contains supplementary material, which is available to authorized users.

## Background

There is now a large number of methods available for prioritizing variants in whole exome or whole genome datasets [1]. These methods commonly identify the variants which are pathogenic, i.e., the variants that may alter normal functions of a protein, either directly through a change in a protein’s amino acid sequence or indirectly through a change of expression [2–4]. In coding and noncoding DNA sequences, there are usually multiple variants that could possibly be pathogenic, but most of them are sub-clinical or will not result in any detectable phenotypic manifestations [5].

Recently, several methods have become available that utilize information about phenotypes observed in a patient to identify potentially causative variants [6–9]. Phenotypes are useful for identifying gene–disease associations because they implicitly reflect interactions occurring within an organism across multiple levels of organisation [10–12]. Phenotype-based methods work by comparing the phenotypes of a patient with a knowledgebase of gene-to-phenotype associations. A measure of phenotypic similarity is computed between a patient’s phenotypes and abnormal phenotypes associated with gene variants or mutations. The phenotypic similarity is then used either as a filter to remove pathogenic variants in genes that are not associated with similar phenotypes to the ones observed in the patient [9] or as a feature in machine learning approaches [6, 7].

The gene-to-phenotype associations used in phenotype-based prioritization strategies come from clinical observations such as those reported in the Online Mendelian Inheritance in Man (OMIM) database [13] or in the ClinVar database [14]. In some cases, they may also come from model organisms. Comparing model organism phenotypes to human phenotypes (i.e., the phenotypes observed in a patient) requires a framework that allows phenotypes of different species to be compared, such as the Uberpheno [15] or PhenomeNET ontology [16].

We have previously developed the PhenomeNET Variant Predictor (PVP) [7] to prioritize causative variants in personal genomic data. We have shown that PVP outperforms other phenotype-based approaches such as the Exomiser or Genomiser tools [17, 18], or Phevor [9]. PVP is based on a random forest classifier, similarly to Exomiser and Genomiser, which also use a random forest. Features used to classify a variant as causative or non-causative combine a phenotype similarity score (to prioritize a gene as being associated with the phenotypes observed in the patient) and a pathogenicity score, as well as other features such as the mode of inheritance and genotype of the variant. As most variants are neutral, there is a very large imbalance between positive and negatives, and the challenges for building a machine learning model for finding causative variants is to account for this imbalance during training and testing.

Recently, deep neural networks have shown to be successful in many domains [19] and often result in better classification performance [4]. We have developed DeepPVP, an extension of the PVP system which uses deep learning and achieves significantly better performance in predicting disease-associated variants than the previous PVP system, as well as competing algorithms that combine pathogenicity and phenotype similarity. DeepPVP not only uses a deep artificial neural network to classify variants into causative and non-causative but also corrects for a common bias in variant prioritization methods [20, 21] in which gene-based features are repeated and potentially lead to overfitting. DeepPVP is freely available at https://github.com/bio-ontology-research-group/phenomenet-vp.

## Implementation

### Training and testing data

We downloaded the ClinVar database 7th Feb, 2017, and extracted GRCh37 genomic variants annotated with at least one disease from OMIM, characterized as *Pathogenic* in their clinical significance, and not annotated with *conflicting interpretation* in their review status. We obtained 31,156 pathogenic variants associated with 3938 diseases in total and the set of these variants constitutes candidate positive instances in our training dataset. We also extracted GRCh37 genomic variants that are characterized as *Benign* in clinical significance, and not annotated with *conflicting interpretation* in their review status. We obtained 23,808 such benign genomic variants from ClinVar which form the candidate negative instances in our training dataset. We excluded any variant records mapped to more than one gene and variant records with missing information on the reference or alternate alleles. For pathogenic variant records, we defined variant–disease pairs for each pathogenic variant and its associated OMIM disease. In our dataset, some pathogenic variants are annotated with multiple OMIM diseases. For each of these variants and the *n* OMIM diseases they may cause, we created *n* variant-disease pairs. For example, variant *rs201108965* in *TMEM216* is annotated with two diseases; Joubert syndrome 2 (OMIM:608091) and Meckel syndrome type 2 (OMIM:603194). We define two variant-disease pairs (*rs201108965*, OMIM:608091) and (*rs201108965*, OMIM:603194). After this step, we have 30,770 pathogenic variant-disease pairs and 20,174 benign variants.

In DeepPVP, we use the zygosity of a variant as one of the training features. The zygosity information is not provided in ClinVar. In a given Variant Call Format (VCF) [22] file, zygosity is represented in the genotype field. For instance, a heterozygous variant will have a genotype value 0/1, while a homozygous variant will have a genotype value 1/1 in the VCF file. We assigned the genotype information to our pathogenic variant-disease pairs based on the mode of inheritance associated with the disease caused by the variant. We extracted the mode of inheritance of the associated OMIM disease using the information provided by the Human Phenotype Ontology (HPO) annotations of OMIM diseases [23]. If the disease’s mode of inheritance is recessive, we assigned the zygosity of the variant as homozygote (denoted with genotype 1/1). In this case, we created a variant-disease-zygosity triple representing this information. If the OMIM disease’s mode of inheritance is not recessive (i.e., any other mode of inheritance, including dominant, unknown, X-linked, etc.), we generated two variant-disease-zygosity triples and characterized one of them as homozygote (denoted with genotype 1/1) and another as heterozygote (denoted with genotype 0/1). For example, pathogenic variant *rs397704705* in *AP5Z1* is associated with Spastic paraplegia 48 (OMIM:613647). This OMIM disease is recessive and, hence, we characterize variant *rs397704705* with genotype 1/1, generating a variant-disease-zygosity triple consisting of variant *rs397704705*, disease OMIM:613647, and genotype 1/1. Another example is the pathogenic variant *rs387907031* in *ARHGAP31* associated with Adams-Oliver syndrome 1 (OMIM:100300). This disease is dominant and, hence, we generated two variant-disease-zygosity triples: variant *rs387907031*, disease OMIM:100300, and the genotype 0/1, and variant *rs387907031*, disease OMIM:100300, and genotype 1/1. Since benign variants are not associated with a disease or mode of inheritance, we treat each of them as both a homozygote and heterozygote, generating two variant-zygosity pairs for each benign variant. After this step, we obtained 61,540 triples consisting of pathogenic variant, disease, and zygosity, and 40,348 pairs of benign variant and zygosity.

The triples consisting of variant, disease, and zygosity constitute positive samples. For each positive instance (*V*,*D*,*Z*) consisting of a variant, disease, and zygosity, we randomly select, with equal probability, one of two possible negative instances: a randomly selected benign variant in the same gene as *V*, or a triple (*V*,*D*^′^,*Z*) where *D*^′^≠*D*. To map intergenic variants to genes, we link variants to their nearest gene.

For example, a positive instance in our training data is a pathogenic variant *rs267606829* in *FOXRED1*, associated with Mitochondrial complex I deficiency (OMIM:252010), as a homozygote. A negative instance according to the first strategy could be a benign variant, such as *rs1786702*, in *FOXRED1*, as a heterozygote. A negative instance according to the second strategy is the same pathogenic variant, *rs267606829* in FOXRED1, as a homozygote, but associated with another OMIM disease such as Tooth Agenesis (OMIM:604625). The resulting training data is balanced.

As an independent and unseen evaluation dataset, we downloaded all variants from ClinVar released between Feb 8th 2017 and Jan 27th 2018. In this dataset, we processed all GRCh37 variants in the same manner as for our training dataset to construct triples of pathogenic variants, disease, and zygosity. However, if the OMIM disease’s mode of inheritance of the variant is not recessive (i.e., any other mode of inheritance, including dominant, unknown, X-linked, etc.), we assigned the zygosity randomly either as homozygote (denoted with genotype 1/1), or heterozygote (denoted with genotype 0/1). We obtained a total of 5686 such triples associated with 1370 diseases for validation.

### Generation of synthetic patients

In our evaluation, we generated a set of synthetic patients as a realistic evaluation case, similarly to previous work [7, 18]. We randomly selected a whole exome from the 1000 Genomes project [24] and inserted a pathogenic variant *V*, assign the disease associated with *V* in ClinVar to the exome, and present *V* as a homo- or heterozygote based on the mode of inheritance associated with the disease. Each of these exomes together with the disease’s phenotypes and mode of inheritance form a synthetic patient in which we aim to recover the inserted variant.

### Annotating variants

Annotating variants with pathogenicy scores from CADD [3], DANN [25], and GWAVA [26] is a time-consuming process in PVP [7] and other phenotype-based variant prioritization tools [18], especially when analyzing WGS data comprised of millions of variants. PVP 1.0 uses tabix [27] for indexing and retrieval of the pathogenicty scores per chromosome and genomic position. To optimize the annotation phase of DeepPVP, we extracted 31,491,995 variants from samples of the 1000 Genomes Project [24] and annotated them with pathogenicity scores from CADD, DANN, and GWAVA. DeepPVP keeps this set of pre-annotated variants in memory to provide fast retrieval of annotations for common variants. DeepPVP utilizes tabix only when the variant annotated is not available in the pre-annotated library, and therefore minimizes disk access.

### Model and availability

We implemented our DeepPVP deep neural network in Python 2.7. We used Keras [28] with a TensorFlow backend [29]. We used one hot encoding to represent our categorical feature of the inheritance mode of the disease. We handled missing values for CADD, GWAVA, DANN, and semantic similarity scores by mean imputation. We also added additional flags for missing values as features. We retrieved gene-phenotype association data from human and model organisms (mouse and zebrafish) on Feb 7th, 2017 and used them to generate the ontology and high level phenotypes and semantic similarity score features.

We used the Hyperas [30] Python library for tuning the hyperparameters of the neural network using the tree-structured Parzen estimator (TPE) algorithm [31]. We selected the following hyperparameters for tuning: number of hidden layers (two, three, or four), number of hidden units in each layer (32, 64, 67, 128, 134, 201, 256, and 512), and the batch size (2500, 5000, 10,000, 15,000, and 20,000). The hyperparameters combination resulting in best performing model out of 50 trials using Hyperas was selected for the final model setup. Therefore, we designed a sequential model with an input layer, three hidden layers of 67, 32, 256 neurons respectively with Rectified Linear Units (ReLU) [32] activation function, and an output layer with a sigmoid activation function. We trained the model using the Adam optimization algorithm [33] which has been widely adopted for deep learning as a computationally efficient, fast convergent, extension to stochastic gradient descent. We used dropout [34] between the hidden layers and the output layer to prevent overfitting. We trained our DeepPVP model for 100 epochs, 2500 batch size, and a learning rate of 0.001. In training, we specified a 20% random stratified-by-disease validation set, and binary cross entropy loss function. We kept the rest of the parameters in their default values. The model was trained on the CPU.

The DeepPVP system (version 2.1), training and evaluation experiments, the synthetic genome sequences and our analysis results can be found at https://github.com/bio-ontology-research-group/phenomenet-vp.

## Results

### DeepPVP: phenotype-based prediction using a deep artificial neural networks

We developed the Deep PhenomeNET Variant Predictor (DeepPVP) as a system to identify causative variants for patients based on personal genomic data as well as phenotypes observed in the patient. We consider a variant to be causative for a disease *D* if the variant is both pathogenic and affects a structure or function that leads to *D*. This distinction is motivated by the observation that healthy individuals can have multiple highly pathogenic variants resulting in a complete loss of function; it is therefore not usually sufficient to identify pathogenic variants alone as there may be many.

DeepPVP is a command-line tool which takes a Variant Call Format (VCF) [22] file as an input together with a set of phenotypes coded either through the Human Phenotype Ontology (HPO) [35] or the Mammalian Phenotype Ontology (MP) [36]. It outputs a prediction score for each variant in the VCF file; the prediction score measures the likelihood that a variant is causative for the phenotypes specified as input to the method.

To predict whether a variant is causative or not, DeepPVP uses similar features as the PVP system [7] and combines multiple pathogenicity prediction scores, a phenotype similarity computed by the PhenomeNET system, and a high-level phenotypic characterization of a patient. The full list of features used by DeepPVP is listed in Additional file 1: Table S1. All features can be generated from a patient’s VCF file and a set of phenotypes coded either with HPO or MP.

**Fig. 1:**
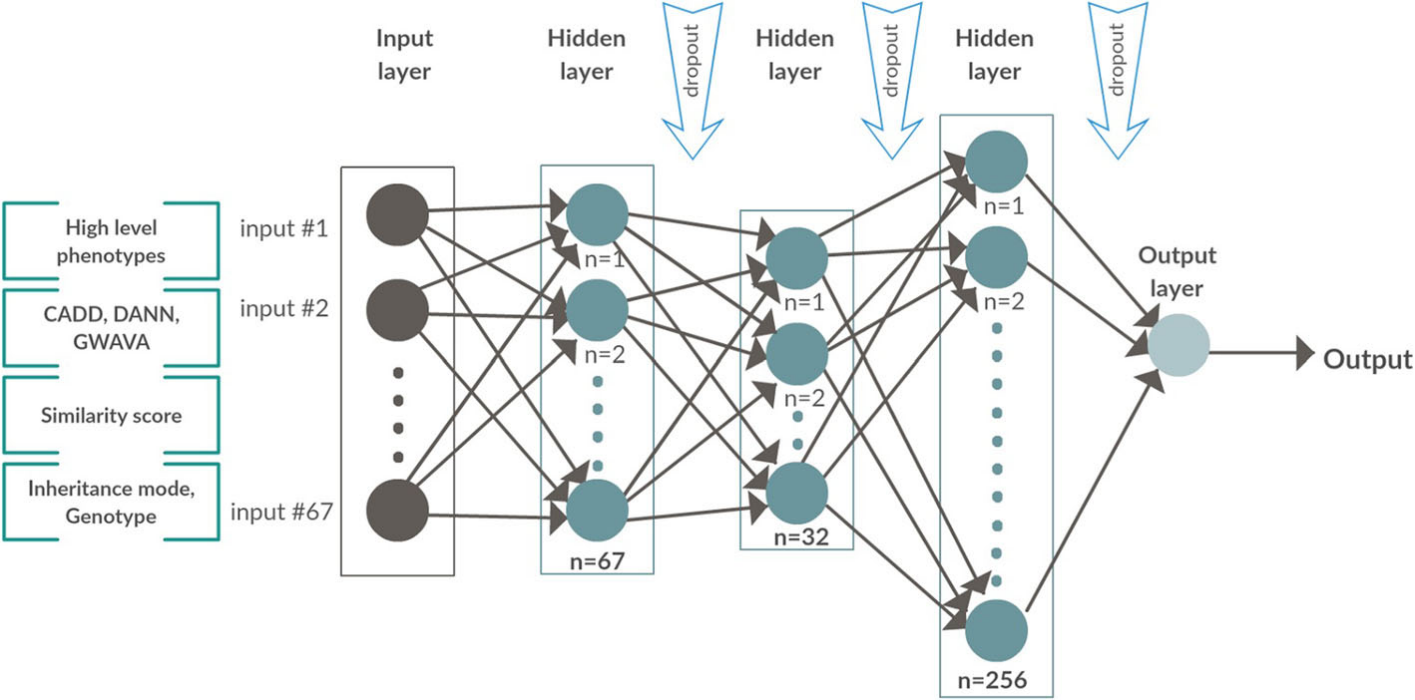
The DeepPVP neural network model

In DeepPVP, we use a deep neural network to classify variants as causative or non-causative. Specifically, DeepPVP uses a feed forward neural network with five layers (see Fig. 1). The input layer in our architecture consists of 67 neurons (for the 67 features) and an output layer consisting of a single output neuron which outputs the prediction score of DeepPVP. DeepPVP uses three hidden layers with 67, 32, and 256 neurons, respectively. Each hidden layer uses a Rectified Linear Unit (ReLU) [32] activation function, and the output layer uses a sigmoid activation function.

DeepPVP is trained similarly as PVP to improve performance of identifying causative variants in real genomic sequences (in contrast to performance on a testing set). When training DeepPVP, we use as positive instances all causative variants from our training set together with the phenotypes of the disease for which they are causative. We discriminate these from two kinds of negatives: benign variants (i.e., variants that do not alter protein function) and pathogenic but non-causative variants. We consider pathogenic non-causative variants as pathogenic variants (in our training set) which are not associated with phenotypes of the disease they cause, but rather with a different disease. The aim of this selection strategy is to discriminate causative variants from all other variants.

We train the DeepPVP model using back-propagation, using binary cross entropy as loss function, and evaluate the model’s results on predicting causative variants, and compare against several competing methods. While the different evaluation scenarios omit some parts of the information about variants and the diseases they are associated with in order to not bias the evaluation results, we finally retrain a model using all available information and make it available as the final DeepPVP prediction model.

### Evaluating DeepPVP’s ability to find causative variants

We use a nested cross-validation experiment as our main evaluation and as means to optimize hyperparameters of our DeepPVP model. We first split our training instances into five folds (80% for training and 20% for testing) where each fold is stratified by disease (i.e., the diseases are disjoint between all five folds). We use the training part in each of these folds for optimizing parameters and hyperparameters and use the 20% to evaluate and report the final performance. In each of the resulting five folds, we split the 80% training set into further five folds that are similarly stratified by disease. In total, we end up with 25 different training sets (in the second level of this nested cross-validation) and evaluation sets.

For each of the 25 different splits of data, we tuned the hyperparameters of the network (number of hidden layers, number of hidden units, and batch size) using the Hyperas library [30]. We generated 50 trial models with 50 epochs for each model using the tree-structured Parzen estimator algorithm [31] to find optimal hyperparameters. We ended up with 25 optimized hyperparameters, and we selected the hyperparameters that resulted in the best performance in most of the folds.

After hyperparameter optimization, we train five different models using the optimal set of hyperparameters obtained in the second level of the cross-validation and evaluate the predictive performance of the model on the 20% set that has not been used for optimizing hyperparameters. The resulting accuracy of our model in 5-fold cross-validation is 0.911, area under the receiver operating characteristic curve (ROCAUC) [37] is 0.959, and the area under the precision–recall curve (AUPR) is 0.954. For comparison, we also performed the same training and evaluation steps using a random forest classifier, and we obtained an accuracy of 0.898, a ROCAUC of 0.958, and an AUPR of 0.952.

To accurately evaluate the performance of DeepPVP on real sequencing data, we apply DeepPVP to causative variants added to the ClinVar database on or after Feb 7th, 2017 while our training data is restricted to the variants that have been added to ClinVar before this date. Between Feb 7th, 2017 and Feb 6th, 2018, there were 5686 causative variants added to ClinVar, covering 1370 diseases. 297 of these diseases were not present in our training data. Evaluation on completely unseen variants allows us to estimate under more realistic conditions how well DeepPVP is able to prioritize novel variants.

We generated synthetic patient exomes by inserting a single causative variant from the set of 5686 variants in a randomly selected exome from the 1000 Genomes Project [24] (removing all variants with Minor Allele Frequency (MAF) greater than 1% (using the frequencies provided by the 1000 Genomes across all populations). We then assign the phenotypes associated with the causative variant in ClinVar, as well as the mode of inheritance of the disease, to the synthetic exome and consider this combination a synthetic patient. We use DeepPVP to prioritize variants given the synthetic patient’s filtered VCF file, phenotypes, and mode of inheritance, and determine the rank at which the causative (inserted) variant is found. For comparison, we use PVP v1.1 [7] as well as the random forest classifier we trained using the same training setup as DeepPVP (named DeepPVP-RF). We further compare the performance to the Exomiser version 7.2.1 released on Feb 6th, 2017 with and without using CADD scores as feature. Furthermore, we compare the performance against CADD [3], DANN [25], and GWAVA [26]. Table 1 shows the evaluation results. We find that DeepPVP has an improved performance compared to the original PVP, the use of a neural network classifier gives better results than the random forest classifier, and DeepPVP outperforms Exomiser, CADD, DANN, and GWAVA in this evaluation.

**Table 1:** Comparison of top ranks of ClinVar variants as recovered from WES data; variants with MAF > 1% are filtered

	Top hit	Top 10 hits	Total	ROC AUC	AUPR
DeepPVP	4060 (71.40%)	4750 (83.54%)	5686	0.94	0.66
DeepPVP-RF	3520 (61.91%)	4321 (75.99%)	5686	0.95	0.55
PVP 1.1	3619 (63.65%)	4076 (71.68%)	5686	0.95	0.55
Exomiser	2910 (51.18%)	3608 (63.45%)	5686	0.89	0.43
Exomiser-CADD	2926 (51.46%)	3621 (63.68%)	5686	0.89	0.43
CADD	1060 (18.64%)	2429 (42.72%)	5686	0.94	0.14
DANN	170 (2.99%)	1322 (23.25%)	5686	0.90	0.03
GWAVA	63 (1.11%)	264 (4.64%)	5686	0.66	0.01

Of the 5686 “new” variants in our ClinVar evaluation set, 5489 are in 934 genes which are associated with phenotypes. These 5489 variants are associated with 1289 diseases. Only 197 variants are in 74 novel genes and are associated with 89 diseases. We test the performance of DeepPVP separately on these 197 variants. DeepPVP identifies 46 of the 197 variants (23%) at rank one, and 87 variants (44%) in the first ten ranks. In comparison, Exomiser and CADD identified 26 and 13 variants at the first, and 61 and 51 variants in the top ten ranks, respectively. Exomiser identified 27 at rank one, and 64 in the top ten ranks. This evaluation demonstrates that DeepPVP can not only identify variants in known disease-associated genes but also in novel genes, although with lower performance than if the gene is already known. While the predictive performance of DeepPVP in this evaluation is lower than in the other types of evaluation, DeepPVP still improves over established methods such as CADD and Exomiser.

Our performance results demonstrate that DeepPVP can identify causative variants with significantly higher recall at rank one and rank ten than several other methods to which we compare, including the original PVP system [7] from which DeepPVP is derived. In some applications of variant prioritization, it is also important to identify causative variants quickly and with low computational costs. We therefore benchmarked the time it takes DeepPVP to process large VCF files. We used a machine equipped with 128 GB Memory and an Intel Xeon ES-2680 v3 CPU with 2.50GHz and 16 cores, using a 64-bit Ubuntu 16.04 system. We selected a genome from the Personal Genome Project (PGP) [38] which contains 4,120,185 variants to benchmark DeepPVP. We prioritized variants in this genome using DeepPVP ten times and recorded the time elapsed. On average, analysis of all the variants in the whole genome VCF file took DeepPVP 85 min, i.e., approximately 1.3 milliseconds per variant. Analyzing the same VCF file using the phenotype-based Exomiser software (with and without CADD annotations) took 189 min without CADD annotations (approximately 2.7 milliseconds per variant) and 800 min with CADD annotations (approximately 11.6 milliseconds per variant).

## Conclusions

DeepPVP is an easy to use and fast phenotype-based tool for prioritizing variants in personal whole exome or whole genome sequence data. DeepPVP takes a VCF file of an individual as input, together with an ontology-based description of the phenotypes observed in an individual. It then aims to identify the variants of the individual that are causative of the phenotypes observed.

Through the use of a deep neural network and an updated training and evaluation strategy, DeepPVP improves over its predecessor PVP, and further outperforms several established methods for variant prioritization, including the phenotype-based tool Exomiser [17, 18] and pathogenicity scoring algorithms such as CADD [3]. Importantly, DeepPVP shows a better performance than other methods in finding variants in novel genes, i.e., genes not previously associated with a disease phenotype, and may therefore be particularly suited for investigating variants in orphan diseases as well as variants of unknown significance in genes not yet associated with phenotypes.

We update DeepPVP in regular intervals when new training data (i.e., variants associated with diseases and phenotypes, as well as gene–phenotype associations) becomes available. DeepPVP is freely available at https://github.com/bio-ontology-research-group/phenomenet-vp.

## Availability and requirements

- Project name: DeepPVP
- Project home page: https://github.com/bio-ontology-research-group/phenomenet-vp
- Operating system: Java virtual machine
- Programming language: Java, Groovy, Python
- Other requirements: none
- License: 4-clause BSD-style license

## Additional file

Additional file 1 Features used to train DeepPVP. A table consisting of the features and their representation used in the training and prediction of DeepPVP. (PDF 33 kb)

## Abbreviations

AUPR: Area under precision-recall curve
CADD: Combined annotation dependent depletion
DeepPVP: Deep PhenomeNet variant predictor
DeepPVP-RF: Deep PhenomeNet variant predictor (random forest)
GWAVA: Genome wide annotation of variants
HPO: Human phenotype ontology
MAF: Minor allele frequency
MP: Mammalian phenotype ontology
OMIM: Online mendelian inheritance in man
PGP: Personal genome project
Phevor: Phenotype driven variant ontological re-ranking tool
PVP: PhenomeNet variant predictor
ReLU: Rectified linear units
ROCAUC: Receiver operating characteristic area under the curve
VCF: Variant call format
WES: Whole exome sequencing
WGS: Whole genome sequencing
