# Supplementary material for: DeepPVP: phenotype-based prioritization of causative variants using deep learning
Source: BMC Bioinformatics. 2019 Feb 6;20:65. doi: 10.1186/s12859-019-2633-8 (PMC6364462; doi:10.1186/s12859-019-2633-8)
Supplement: Supplementary file 1 — Features used to train DeepPVP. A table consisting of the features and their representation used in the training and prediction of DeepPVP. (PDF 33 kb) [file 12859_2019_2633_MOESM1_ESM.pdf]

Table 1: DeepPVP model features.

Feature	Type
Pathogencity prediction	CADD score (numeric)
	GWAVA score (numeric)
	DANN score (numeric)
Phenotype-related	PhenomeNet similarity score (numeric)
	Disease inheritance mode: Dominant, Recessive, X-linked, Others/Unknown (one-hot encoding)
	High-level phenotype HP_0000078(binary)
	High-level phenotype HP_0000291(binary)
	High-level phenotype HP_0000708(binary)
	High-level phenotype HP_0001001(binary)
	High-level phenotype HP_0001939(binary)
	High-level phenotype HP_0002086(binary)
	High-level phenotype HP_0006476(binary)
	High-level phenotype HP_0009126(binary)
	High-level phenotype HP_0010515(binary)
	High-level phenotype HP_0010948(binary)
	High-level phenotype HP_0010987(binary)
	High-level phenotype HP_0011017(binary)
	High-level phenotype HP_0011025(binary)
	High-level phenotype HP_0011277(binary)
	High-level phenotype HP_0011482(binary)
	High-level phenotype HP_0011915(binary)
	High-level phenotype HP_0040063(binary)
	High-level phenotype MP_0000003(binary)
	High-level phenotype MP_0000358(binary)
	High-level phenotype MP_0000428(binary)
	High-level phenotype MP_0000462(binary)
	High-level phenotype MP_0000516(binary)
	High-level phenotype MP_0000685(binary)
	High-level phenotype MP_0000716(binary)
	High-level phenotype MP_0001188(binary)
	High-level phenotype MP_0001213(binary)
	High-level phenotype MP_0001270(binary)
	High-level phenotype MP_0001533(binary)
	High-level phenotype MP_0001663(binary)
	High-level phenotype MP_0001672(binary)
	High-level phenotype MP_0001764(binary)
	High-level phenotype MP_0001790(binary)
	High-level phenotype MP_0001983(binary)
	High-level phenotype MP_0002060(binary)
	High-level phenotype MP_0002089(binary)
	High-level phenotype MP_0002095(binary)
	High-level phenotype MP_0002106(binary)
	High-level phenotype MP_0002109(binary)
	High-level phenotype MP_0002138(binary)
	High-level phenotype MP_0002139(binary)
	High-level phenotype MP_0002163(binary)
	High-level phenotype MP_0002164(binary)
	High-level phenotype MP_0002396(binary)
	High-level phenotype MP_0003385(binary)
	High-level phenotype MP_0004133(binary)
	High-level phenotype MP_0004134(binary)
	High-level phenotype MP_0005408(binary)
	High-level phenotype MP_0005451(binary)
	High-level phenotype MP_0005621(binary)
	High-level phenotype MP_0009389(binary)
	High-level phenotype MP_0010678(binary)
	High-level phenotype MP_0010769(binary)
	High-level phenotype MP_0012719(binary)
	High-level phenotype MP_0013328(binary)
Genotype	Homozygote or heterozygote (binary)
Imputation Flags	CADD flag (binary)
	GWAVA flag (binary)
	DANN flag (binary)
	PhenomeNet similarity flag (binary)
